# Movable Kidney—I

**Published:** 1908-03-07

**Authors:** 


### Points in Surgery.

### MOVABLE KIDNEY—I.

By the term "movable kidney" one means to imply that there is a greater amount of mobility of the organ than is normally found to be present. For there is always some movement of the kidney in perfectly healthy individuals, which can be explained on physiological grounds. The downward movement of the diaphragm, which accompanies the inflation of the lungs during inspiration, increases the intra-abdominal tension. As a direct result the organs in contact with it, particularly the liver, move downwards; and indirectly all the abdominal viscera are influenced as well. It is even said that the fundus uteri reacts to every respiratory movement. The difficult question to decide is, when does the amount of movement cease to be physiological and become pathological. Normally, even in deep inspiration, the lower pole of the kidney can only just be felt, and then only in a moderately thin patient, who can keep his abdominal walls relaxed. In an obese patient, who resists examination, it is well nigh impossible to do this. The method of examination which is usually advised is to stand on the side of the patient on which the kidney is suspected, and to place one hand on the lumbar region of the anterior abdominal wall, pressing gently with the fingers beneath the margin of the costal arch while the other exerts pressure in the region of the kidney behind. There is, however, another method which is simple, and has some advantages. The surgeon stands on the right side of the patient, and, leaning over him, grasps one flank in each hand, the fingers being behind, and the thumb in front. Steady gentle pressure will enable him, in a tractable patient, almost to approximate the anterior and posterior abdominal walls between the costal arch and the crest of the ilium. Now if the patient takes a deep breath the lower pole of each kidney may be felt. This method has the advantage that it is more certain than the usual system of examination, and further that the mobility of both kidneys can be examined at the same time, and their relative positions compared. Allowance must be made for the fact that the right kidney lies normally somewhat lower in the abdomen than does the left. It is a mistake to give an anæsthetic for this examination, as is sometimes done: the success of the method depends upon persuading the patient to take a deep inspiration, and under the anæsthetic he is, of course, unable to do this.

The term "movable kidney" should be reserved for those cases in which the organ only moves in one plane, the vertical. This has been compared to a "cinder-sifting" movement; and this again may be sub-divided into (*a*) palpable kidney in which the lower pole of the kidney can be fixed between the thumb and fingers, and (*b*) movable kidney proper when the upper pole can be distinctly palpated. Advanced cases in which the kidney moves in the antero-posterior as well as in the vertical plane, and may be found in any part of the upper abdomen, should be classified under the heading of "floating kidney."

The causation of this condition has given rise to no little speculation; but investigation is rendered difficult by the almost constant association of a greater or lesser degree of neurasthenia, so that it is hard to know whether the kidney is really the cause of the symptoms or whether they are simply one of the many manifestations of the nervous condition. There seems to be little doubt that genuine cases are more common in women than in men; and this is explained on the ground that the paravertebral groove is shallower in them. How far abnormalities of, or deficiencies in, the normal anatomy of the kidney capsule, or of its relations to surrounding parts can be accused of producing the condition is not known. It seems, however, that the kidney is not normally suspended from the posterior abdominal wall by a capsule, but it is rather kept in position by the mutual pressure of the organs on one another in the abdominal cavity. In any case it is moderately certain that anything which weakens the abdominal parietes modifies the maintenance of the normal intra-abdominal pressure and deprives the viscera of their accustomed support. Parturition may lead to this result, and the removal of large abdominal tumours not infrequently does so. It is hard to explain why the kidney should be more affected in this way than other organs. It is true that in some cases a movable kidney is only a small part of the condition, and that in reality all the abdominal organs have shifted their positions. Enteroptosis or Glenard's disease is a well recognised clinical entity. In this the contents of the abdomen tend to assume a lower position than they normally should, particularly the liver and the stomach; and if these organs are misplaced, it is only reasonable to suppose that the kidneys, which are in direct contact with them, will correspondingly alter their position. In other cases a severe injury has been thought to have been the primary cause. One definite case was reported during the South African War. An officer was thrown from his horse and fell in a sitting position on his tubera ischiorum. He had previously had excellent health, but subsequently suffered from more or less severe abdominal pain. Examination showed that both kidneys were definitely displaced, the upper pole being easily palpated on either side. Curiously enough, he developed later symptoms of neurasthenia to a marked degree, and complained of a number of troubles, which could not be attributed to the renal condition. This is unusual because, although neurasthenia is commonly associated with a movable kidney, it is rare for a man previously healthy to become a hypochondriac as the direct result of the kidney becoming movable. What usually happens is that a neurasthenic patient complaining of vague pains seeks advice from one medical man after another until one of them, in an evil moment, discovers that the kidney is movable, and informs the patient of the fact. From that moment his attention is focussed on that organ, and all his symptoms are attributed to it.

